# Parent–adolescent communication on sexual and reproductive health issues and associated factors among secondary public-school students in Gondar town, northwest Ethiopia: an institution based cross-sectional study

**DOI:** 10.3389/fpubh.2024.1342027

**Published:** 2024-08-19

**Authors:** Mihret Melese, Dereje Esubalew, Tsehayu Melak Siyoum, Yilkal Belete Worku, Jember Azanaw, Berihun Agegn Mengistie

**Affiliations:** ^1^Department of Human Physiology, School of Medicine, College of Medicine and Health Sciences, University of Gondar, Gondar, Ethiopia; ^2^Department of Human Physiology, College of Medicine and Health Sciences, Ambo University, Ambo, Ethiopia; ^3^Department of Neonatal Nursing, School of Nursing, College of Medicine and Health Sciences, University of Gondar, Gondar, Ethiopia; ^4^Department of Internal Medicine, School of Medicine, College of Medicine and Health Sciences, University of Gondar, Gondar, Ethiopia; ^5^Department of Environmental and Occupational Health and Safety, College of Medicine and Health Sciences, Institute of Public Health, University of Gondar, Gondar, Ethiopia; ^6^Department of General Midwifery, School of Midwifery, College of Medicine and Health Sciences, University of Gondar, Gondar, Ethiopia

**Keywords:** parent–adolescent communication, sexual and reproductive health issues, students, secondary schools, Ethiopia

## Abstract

**Introduction:**

Effective communication between adolescents and their parents is crucial for reducing sexual health problems. This open dialogue can help address misconceptions, provide accurate information, and foster a supportive environment where adolescents feel comfortable seeking guidance and discussing sensitive issues related to their sexual health. In Ethiopia, with its diverse ethnic and cultural background, effective communication between parents and adolescents about sexual and reproductive health (SRH) is crucial in reducing the likelihood of adolescents engaging in risky sexual behaviors. Despite the importance of such communications, there were no data showing the level of parent–adolescent communication (PAC) in secondary public schools in Gondar town. Therefore, this study aimed to determine the level of parent–adolescent communication on sexual and reproductive health issues along with its influencing factors, among secondary students in Gondar town, northwest Ethiopia.

**Methods:**

We employed an institution-based cross-sectional study design. A total of 424 students were recruited using a systematic random sampling technique, with a 100% response rate. We developed a structured questionnaire from the related literature to collect data from the participants of the study. The data were entered using EpiData version 4.6, and analyzed using SPSS version 25. A binary logistic regression model was fitted to identify associated factors.

**Results:**

The proportion of adolescents who had communicated with their parents was 37.7% (95% CI: 34.65–44.76). In a multivariable analysis at a 95% confidence interval (CI), variables such as being female (adjusted odds ratio (AOR) = 2.23; 95% CI: 1.09–7.45), belonging to grades 11–12 (AOR = 1.25; 95% CI: 1.19–6.98), living with parents/caregivers (AOR = 1.26; 95% CI: 1.07–5.66), having a positive attitude toward sexual health (AOR = 2.4; 95% CI: 1.34–7.82), having poor knowledge about SRH issues (AOR = 1.23; 95% CI: 1.04–7.81), and having good knowledge about the puberty period (AOR=1.23; 95% CI:1.04–7.89) were statistically associated with parent–adolescent communication.

**Conclusion and recommendations:**

This study found a low level of communication between parents and adolescents regarding sexual and reproductive health (SRH) issues. To address this challenge, it is crucial to implement evidence-based education on SRH topics, such as consent, healthy relationships, communication skills, STDs, contraception, and interpersonal dynamics. Enhancing parent–adolescent dialogue on SRH can be achieved by implementing peer education among senior students and training teachers in effective communication techniques. The study also recommended conducting qualitative research to explore the specific barriers affecting parent–adolescent communication.

## Introduction

Poor communication skills between parents and adolescents regarding sexual and reproductive health (SRH) can lead to misinformation, misunderstandings, and a reluctance to seek help (1–3). The level of parent–adolescent communication on sexual and reproductive health issues varies across different countries in developed regions. For instance, it was reported to be highest in the United States of America at 70.6% and in Mexico at 83.1%. Conversely, lower levels of parent–adolescent communication were observed in Myanmar at 6.8% and in India at 13% (4–7). Similarly, the prevalence of parent–adolescent communication was reported to be lower in African countries. For instance, in Nigeria, it was reported as 37.4% (8), while in Lesotho, the prevalence was 20 % (9). In Ethiopia, the prevalence was reported to be within the range of 25.3% and 36.9% (10). Parent–adolescent communication regarding sexuality is critical in informing young people about risks and protective behaviors, which, in turn, decreases the likelihood of involvement in risky sexual behaviors (11, 12).

Factors that affect parent–adolescent communication concerning sexual and reproductive health (SRH) issues include cultural taboos, embarrassment in discussing sexual matters, lack of communication skills, beliefs about sexuality, and knowledge gaps (13, 14).

Adolescents are often underserved by current health services, which highlights the importance of prioritizing their needs in universal health coverage initiatives after 2015 (15). Many adolescents die prematurely due to preventable or treatable causes, including accidents, suicide, violence, pregnancy complications, and reproductive illnesses (16, 17). According to the WHO reports, 1.3 million young people die each year from preventable causes (18). In sub-Saharan Africa, 82% of the 2.1 million adolescents are affected with HIV, with 58% being female individuals. Comprehensive knowledge about HIV, condom use, testing, and treatment remains low in the poorest countries (16, 18, 19). In addition, approximately 16 million women aged 15–19 years give birth annually, with 95% of these births occurring in low- and middle-income countries (17, 18). Parents who openly discuss sexuality with their young children foster better communication, helping to reduce risky behaviors, such as early sexual initiation, unwanted pregnancies, and other reproductive health problems (13, 15). Effective parent–adolescent communication is important to reduce adolescents' engagement in risky sexual behaviors (13, 14). Discussions between parents and adolescents about sexual and reproductive health enhance awareness, reduce risky behaviors, and promote positive SRH outcomes (20–25). Various studies have indicated that factors such as parents' reluctance to discuss, feelings of shame, cultural taboos, lack of communication skills, limited awareness, and the belief that discussions might encourage sexual activity are key elements that affect parent–adolescent conversations on SRH topics (26–28). In sub-Saharan African countries, including Ethiopia, evidence shows that a lack of parental interest in discussions, feelings of shame, and cultural taboos around discussing sexual matters are factors affecting parent–adolescent communication (29, 30).

In the Ethiopian context of ethnic and cultural diversity, effective parent–adolescent communication is crucial to reduce adolescents' engagement in risky sexual behaviors (10, 31). However, there is limited information on parent–adolescent communication among adolescents attending secondary schools. Hence, the primary objective of this study was to assess the level of parent–adolescent communication and identify associated factors. The research sought to provide valuable insights that could inform strategies to enhance SRH communication, thereby contributing to better health outcomes for adolescents in Gondar town.

## Methods and materials

### Study design

An institution-based cross-sectional study design was employed.

### Study area and period

The study was conducted in secondary public schools located in Gondar town, spanning from 18 August 2023 to 20 September 2023. Gondar town is located approximately 728 km away from Addis Ababa, the nation's capital. In addition, it is situated approximately 180 km away from Bahir Dar, which serves as the capital of the Amhara regional state. Moreover, Gondar town has 22 kebeles and 14 high schools, including 9 government schools and 5 private schools (32).

### Source population

The source population comprised all regular students aged 10–19 years who were attending high schools and preparatory schools in Gondar town during 2023. The study population included students who met the specified inclusion criteria.

### Inclusion criteria

All regular students aged 10–19 years who were attending secondary schools in Gondar town at the time of data collection were included.

### Exclusion criteria

Adolescents who were critically ill or had mental disabilities were excluded from the study.

### Sample size determination

The sample size was calculated using a single population proportion formula. This calculation was based on the assumption that the proportion of parent–adolescent communication on sexual and reproductive health issues is 50% (i.e., *p* = 0.5), with a 95% confidence level and a 5% margin of error.


(1) $$\begin{array}{l} \text{n}=\frac{\left(\frac{za}{2}\right)2^{*}\left(p^{*}\left(1-p\right)\right)}{d2}=\frac{{\left(1.96\right)}^{2}\;0.5\left(1-0.5\right)}{{\left(0.05\right)}^{2}}=384.16\approx \;385 \end{array}$$

Finally, a 10% non-response rate = 39 + 385 = 424 was considered.

### Sampling technique and procedure

**Figure 1:**
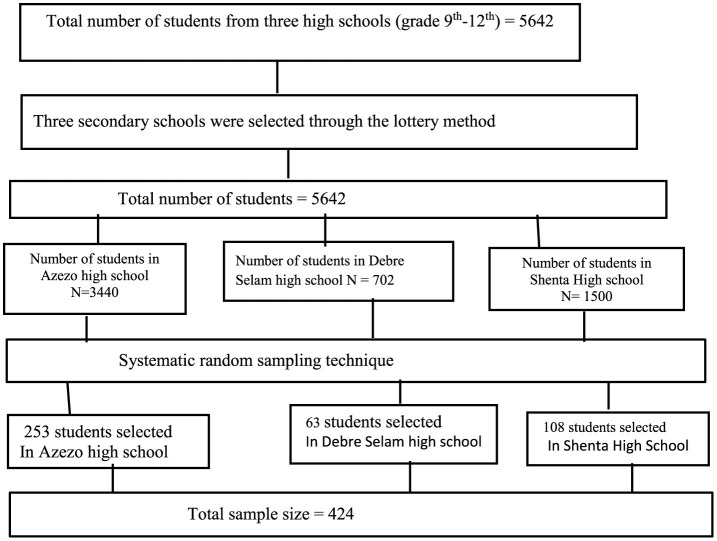
Sampling procedure for secondary school students in Gondar, northwest Ethiopia, 2023.

This study included three public secondary schools (Arezo, Debre Selam, and Shinta). These schools were chosen using a lottery method, and proportional allocation was then applied to each school. To select students for the study, a sampling interval (k) was determined and used for sampling. This interval was calculated by dividing the total number of students in the selected schools (5,642) by the desired sample size (424), which resulted in an interval of 14. Using this sampling interval, we systematically selected every 14th student from the roster books (refer to Figure 1).

### Data collection tool and quality procedures

The data were collected using a pretested, structured, interviewer-administered questionnaire. The questionnaire was initially prepared in English, then translated into the local language (Amharic), and subsequently back-translated into English by language experts to ensure consistency in word meanings. It covered sociodemographic characteristics and sexual reproductive health issues. Data collectors received 2 days of training on the study's objectives, sampling procedures, questionnaire administration, and ensuring questionnaire completeness. Confidentiality was maintained by omitting participants' names from the questionnaire.

### Study variables

#### Dependent variables

Parent–adolescent communication (Yes/No).

The study assessed parent–adolescent communication (PAC) regarding sexual and reproductive health (SRH) issues by assessing the following components: condom use, STIs/HIV/AIDS, sexual intercourse, menstruation, unwanted pregnancy, contraception, and physical and psychological changes during puberty. If the students discussed at least two of the SRH topics with their parents/caregivers in the past 12 months, the communication was marked as “yes”. If not, it was marked as “no”. This approach has been utilized in similar studies (33, 34).

#### Independent variables

The independent variables included the students' grade level, number of children, attitudes and knowledge about sexual and reproductive health issues, and students' religion.

### Operational definitions of the dependent and independent variables

#### Adolescents

All individuals in the 10–19 year age group were defined as adolescents (35).

#### Parents

Parents were defined as individuals who play a significant role in an adolescent's life and provide unpaid care for their work, including biological parents (mother and father), grandparents, older relatives, and other caregivers (36).

#### Knowledgeable about SRH

Students who scored above the mean score on the knowledge questions regarding sexual and reproductive health (SRH) were categorized as knowledgeable, while those who scored below or equal to the mean score were categorized as not knowledgeable (37, 38).

#### Perception of students regarding SRH

We assessed the students' perceptions of discussions on sexual and reproductive health using open-ended questions rated on a Likert scale. The perception index was established, ranging from 0 to 11, with a median score of 9. High perception was defined as scores at or above the median score, while low perception was defined as scores below the median score (39, 40).

### Data processing and statistical analysis

The collected data were coded and entered into EpiData version. Subsequently, the data were analyzed using IBM SPSS Statistics version 25.0 software. Summary statistics, such as proportions and frequencies, were used to represent the results. Bivariable and multivariable logistic regressions were conducted to identify factors associated with the outcome variable. In the bivariable logistic regression, variables with a *p* ≤ 0.2 were included in the multivariable logistic regression model. In this model, variables significantly associated with parent–adolescent communication were identified at a *p* ≤ 0.05. The normality of continuous data was assessed using the Shapiro–Wilk test, and the model's fitness was evaluated using the Hosmer–Lemeshow goodness-of-fit test. The validity of the assessment tool was verified using Cronbach's alpha, which yielded a reliability coefficient of 0.75.

### Ethical consideration

The study received ethical approval from the Institutional Review Board of the University of Gondar (IRB 416/2023). After the approval of the proposal, an official letter was written to the Gondar town administration to request permission and support. In addition, permission was secured from the school administration and the school parent–teacher committee. Before participation, each individual provided informed verbal consent or assent after receiving a detailed explanation of the study's purpose. The confidentiality of the information was strictly maintained.

### Background profile of the study participants

In this study, 424 adolescents were included, with a 100% response rate. The mean age of the adolescents was 16 (±1.73) years. A significant portion, comprising 68.4%, were enrolled in grades 9–10. Out of the total adolescents surveyed, nearly half (48.1%) of them identified as orthodox religious followers. Furthermore, the majority (55.7%) of adolescents reported that they live with their parents or other family members (Table 1).

**Table 1:** Sociodemographic Characteristics of the study's participants in Gondar town public secondary schools, northwest Ethiopia, 2023.

**Variables**	**Category**	**Frequency**	**Percentage**
Sex	Male	244	57.5
	Female	180	42.5
Participant's age (years)	11–15	132	31.4
	16–18	291	68.6
Student's grade	Grade 9–10	290	68.4
	Grade 11–12	134	31.6
Residence	Urban	313	73.8
	Rural	111	26.2
Religion	Orthodox	204	48.1
	Muslim	94	22.2
	Catholic	78	18.4
	Protestant	48	11.3
Living condition	With parents or relatives	237	55.9
	Living alone	187	44.1
Number of parents having male adolescents	Yes	284	67
	No	140	33

### Knowledge of the adolescents on sexual and reproductive health issues

Nearly 73% of the study participants correctly identified the typical onset of puberty. Among the female respondents, an impressive 89.4% accurately recognized the onset of the puberty period. More than half of the adults responded correctly regarding the development of secondary sex organs during puberty. The majority of the female respondents, 72%, responded correctly regarding the growth of sexual organs during the puberty period. Approximately two-thirds, i.e., 66%, of the adult study participants correctly acknowledged that experiencing attraction toward the opposite sex during puberty is considered normal. Among the respondents who responded correctly regarding this aspect, approximately 64% were categorized as adult adolescents. Out of all the adolescents surveyed, a significant majority of 72% was aware of the average age at which girls typically begin menstruating. It was astonishing how well-informed the majority of the female respondents were on this issue. Over two-thirds of the adults were knowledgeable about the typical duration of menstrual bleeding. Impressively, approximately 95% of the female respondents were informed about this aspect. Nearly 73% of the students were knowledgeable about contemporary contraception methods, while over 95% of the female respondents were well-informed on the subject.

The majority of the student participants (75%) were acquainted with information about safe abortion practices. Moreover, over 80% of the female respondents demonstrated familiarity with the topic of safe abortion (Table 2).

**Table 2:** Knowledge about sexual and reproductive health issues among the adolescent students in Gondar town government secondary schools, northwest Ethiopia, 2023.

**Variables**	**Correct response**		
Knowing the changes that occur on a physical and emotional level during adolescence		N=244	N=180
	Overall	Male *N* (%)	female*N* (%)
Puberty normally begins in boys aged 12–16 years and in girls aged 10–14 years	309 (72.8%)	148 (60.6%)	161 (89.4%)
Growth of sex organs during puberty, including the development of hips and breasts in girls and enlargement of the penis and testicles in boys	230 (54.2%)	100 (41%)	130 (72%)
Both sexes develop pubic and underarm hair during adolescence.	140(33%)	85 (35%)	55 (30.6%)
The body's production of the hormone testosterone encourages men to ejaculate or release sperm and have erections.	145 (34.2)	77 (31.6)	68 (37)
It is normal for adolescents to feel sexually aroused.	270(63.7%)	160 (65.6%)	110(61%)
Attraction toward the opposite sex is normal during puberty	280 (66%)	157 (64.3%)	123 (68.3%)
Adolescents frequently experience wet dreams or nocturnal emissions.	163 (38.4%)	120 (49.2%)	43 (23.9%)
During puberty, glands in the skin of the back, shoulders, and face begin to activate more, producing more oil.	274 (64.6%)	132(54%)	142 (78.9%)
**Knowing information about menstruation**			
Knowing the average age at which girls begin to menstruate	308 (72.6%)	160 (65.65)	148 (82%)
Knowledge about the typical menstrual bleeding duration	289 (68%)	119 (48.8%)	170 (94.4%)
Knowledge about absorbing products that are best used when a woman is menstruating	270(63.7%)	120 (49.2%)	150(83%)
Girls can go to school during menstruation	268 (63.2%)	109 (44.6%)	159 (88.3%)
Knowing that having a period causes it to smell bad	229 (545)	89 (49.4%)	140 (57.3%)
Knowing that it smells bad when you are menstruating	259 (61%)	134 (55%)	125 (69.4%)
Girls experience menstruation once every 1–4 weeks.	123(295%)	56(23%)	67 (37.2%)
Knowing the items for personal hygiene used during menstruation	178 (72%)	78 (43.3%)	100 (55.6%)
**Knowledge about pregnancy prevention, contraceptives, and safe abortion**			
Knowledge about the contemporary methods of contraception	308 (72.6%)	136 (55.7%)	172 (95.5%)
Knowledge about the availability of safe abortion	318 (75%)	114(46.7%)	146 (81%)
Using a condom for every act of sexual intercourse prevents pregnancy	276 (65%)	153 (62.75)	123 (68.3%)
Pregnancy can be avoided by refraining from sexual activity during the fertile period.	159 (37.5%)	62 (25%)	97 (53.9%)
**Knowledge about sexually transmitted diseases**			
Having no sex at all completely prevents STIs.	318 (75%)	209 (85.6%)	109 (60.55%)
STIs can be avoided by using condoms during all sexual activities.	360 (84.9%)	213(87.3%)	149 (82.7%)
Having sex with an infected individual can spread STIs.	289 (68%)	139(57%)	150 (83.3%)
STIs can spread when sharp objects are shared.	271 (64%)	136 (55.7%)	135(75%)
Contaminated blood transfusion can transmit STIs.	304 (71.7%)	189(77.5%)	115 (63.9%)
Having sex with sex workers increases the risk of STIs.	182(42.9%)	110 (45%)	70(38.9%)
Using the restroom together can spread STIs.	290 (44.8%)	166 (68.4%)	124 (68.9)
STIs can be spread by kissing.	289 (68%)	140 (57%)	149(82.8%)

### Frequently perceived barriers by adolescents in communicating about sexual reproductive health with their parents

A significant hindrance to initiating conversations about sexual health with adolescents stems from feelings of shame or embarrassment among parents, as reported by 52.8% of them. Furthermore, adolescents face several challenges when trying to communicate with their parents about sexual and reproductive health (SRH). These obstacles include concerns about social stigma or fear of social rejection (41%), doubts regarding the accuracy of SRH information or a perceived lack of knowledge (44.6%), apprehension that openly discussing SRH issues may encourage sexual activity (46.2%), and the perception of cultural barriers (30.4%) (Table 3).

**Table 3:** Frequently perceived barriers by adolescents in communicating about sexual reproductive health with their parents in Gondar town government secondary schools, northwest Ethiopia, 2023.

**Frequently perceived barriers by adolescents**	**Correct response**
Feeling uncomfortable or embarrassed	224 (52.8%)
Fear of social exclusion or social stigma	174 (41%)
Feelings of cultural barriers	129 (30.4%)
Feeling that the information is incomplete or inaccurate regarding sexual and reproductive health	189 (44.6%)
Risk perception for engaging in sexual activity following candid discussions on sexual and reproductive health issues	196 (46.2%)
Feeling unable to adequately describe the problems with sexual and reproductive health	143 (33.7%)
Adolescents may not want to talk about sexual health	111 (26.2%)

### Factors associated with parental communication regarding sexual and reproductive health with adolescents

These factors included sex, religion, student grade level, number of adolescent children, living arrangements for adolescents, students' knowledge about puberty, students' knowledge about sexual and reproductive health (SRH) issues, attitudes toward SRH, and knowledge about SRH services at health facilities. However, in the multivariable logistic regression analysis, several factors were significantly associated with parent–adolescent communication on SRH issues, with a *p* ≤ 0.05. These factors included sex, student grade level, living arrangements, students' knowledge about puberty, students' knowledge about SRH communication, and students' attitudes toward sexual and reproductive health (SRH). The odds of parent–adolescent communication were higher among the female adolescents compared to male adolescents (adjusted odds ratio (AOR) = 1.23; 95% confidence interval (CI): 1.09, 7.45). The students in grades 11–12 had higher odds of engaging in parent–adolescent communication on SRH compared to those in grades 9–10 (AOR = 1.25; 95% CI: 1.19–6.98).

The students who lived with their parents or caregivers had higher odds of having parent–adolescent communication compared to those who lived alone (AOR = 1.26; 95% CI: 1.07, 5.66). The students with good knowledge about puberty had higher odds of engaging in parent–adolescent communication compared to those with poor knowledge (AOR = 1.23; 95% CI: 1.04, 7.89). The students who had positive attitudes toward sexual reproductive health issues were more likely to have good parent–adolescent communication compared to those with negative attitudes (AOR = 2.4; 95% CI: 1.34–7.82). Furthermore, students with poor knowledge about sexual reproductive health issues were less likely to engage in parent–adolescent communication compared to those with good knowledge about these issues (AOR = 0.21; 95% CI: 0.13, 0.45) (Table 4).

**Table 4:** Factors associated with parent–adolescent communication regarding SRH among secondary school students in Gondar town, northwest Ethiopia, 2023.

**Variables**	**Category**	**Parents' communication regarding SRH**		**OR (95% CI)**	**AOR (95% CI)**
		Yes	No		
Sex	Female	90 (50%)	90 (50%)	2.42 (1.16–15.44)	2.23 (1.09–7.45)^*^
	Male	70 (29%)	170 (71%)	1	1
Religion	Orthodox	70 (43.8%)	90 (56.2%)	12.66 (4.22–19.56)	7.42 (0.56–12.33)
	Muslim	50 (41.7%)	70 (58.3%)	7.87 (3.77–16.67)	0.87 (0.12–12.33)
	Protestant	30 (33.3%)	60 (66.7%)	4.76 (1.34–8.67)	1.65 (0.65–7.89)
	Catholic	10 (18.5%)	44 (81.5%)	1	1
Adolescents' grade	Grade 9–10	80 (57.9%)	110 (42.15)	1	1
	Grade 11–12	80 (34.1%)	154 (65.9%)	1.40 (1.06–6.87)	1.25 (1.19–6.98)^*^
Adolescents' living conditions	Living with parents	100(40%)	148 (60%)	1.3(1.05–8.45)	1.26 (1.07–5.66)^*^
	Living alone	60(34%)	116 (66%)	1	1
Students' knowledge about the puberty period	Good	104(41%)	150 (59%)	1.41 (1.02–8.76)	1.23 (1.04–7.89)^*^
	Poor	56 (32.9%)	114 (67.1%)	1	1
Students' knowledge about SRH communication	Good	54 (28.6%)	135 (55.4%)	1	1
	Poor	106 (55.5%)	60 (60.8%)	0.24 (0.08–0.87)	0.21 (0.13–0.45)^*^
Students' attitude toward SRH	Positive	130 (47.3%)	145 (52.7%)	3.55 (1.35–12.45)	2.4 (1.34–7.82)^*^
	Negative	30 (20%)	119 (80%)	1	1
Adolescents' knowledge about SRH services at health facilities	Ye	98 (37%)	168 (63%)	1	1
	No	62 (39%)	96 (61%)	0.94 (0.032–0.99)	0.76(0.08–1.89)

^*^p-value < 0.05. SRH, sexual reproductive health.

## Discussion

Adolescents who engage in risky sexual behaviors have a risk of contracting STIs, unintended pregnancies, and delayed health care. Parent–adolescent communication on sexual and reproductive health helps mitigate these risks. This study assessed communication levels between adolescents and parents on these topics and identified associated factors among secondary school students in Gondar town, northwest Ethiopia.

**Figure 2:**
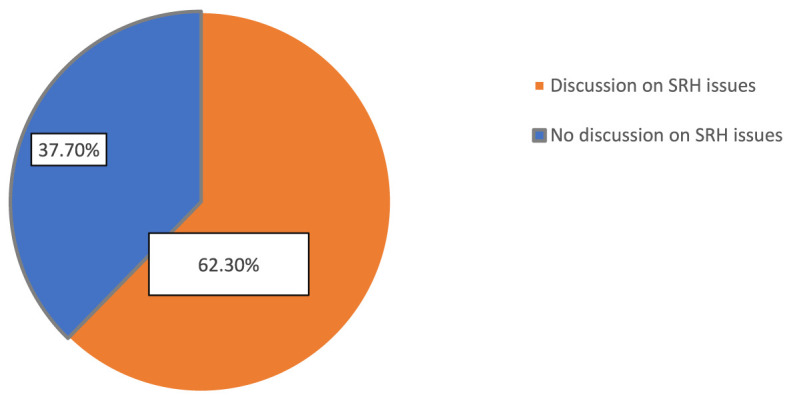
Distribution of parent–adolescent communication on SRH issues in Gondar town, northwest Ethiopia, 2023 (n = 424).

This study found that 37.7% of the adolescents communicated with their parents or caregivers about sexual and reproductive health issues in the past 12 months (95% CI: 34.65–44.76) (Figure 2). This result is consistent with the results from similar investigations carried out in Debre Markos, Northwest Ethiopia (36.9%) (41), Amhara region of Ethiopia (37.5%) (42), and Nepal (40.9%) (43). However, this study's result is lower than the 56.9% reported in Central Ethiopia and Woldia Town (46), and the 56.3% in Dabat Town (46), but higher than the 21.3% found in Assela, Oromia (45). The differences in results stem from socioeconomic factors, cultural norms, and access to sexual and reproductive health (SRH) information. Socioeconomic status affects resources, such as education and healthcare, impacting parental involvement in adolescent health discussions. Cultural beliefs influence attitudes toward discussing SRH, with variations in openness across different cultures. Access to SRH information also varies by region, influencing adolescents' knowledge and comfort level in discussing these topics with parents. Regions with better SRH education tend to have more parent–adolescent communication on these issues (47, 48).

In this study, parent–adolescent communication was significantly associated with several factors: sex, student grade level, living arrangements, students' knowledge about puberty, their understanding of SRH communication, and their attitudes toward sexual and reproductive health (SRH). Female students tended to have higher levels of communication with their parents about SRH compared to male students, which is consistent with the findings from other studies (47, 49, 50). This might be attributed to factors such as female adolescents being generally more open to discussing sensitive topics with their parents and feeling more comfortable seeking guidance and information from their parents regarding SRH concerns (25, 50–53). Moreover, female adolescents often have more opportunities to spend time at home, which could facilitate discussions with their parents on these matters. In addition, parents or caregivers may feel more compelled to communicate with or provide guidance to their daughters regarding SRH topics as a means of preventing premarital sexual activity, unintended pregnancies, and induced abortions among female adolescents. Ultimately, effective and open parent–adolescent communication about SRH issues are equally crucial for both sexes (20, 54, 55).

It was found that the students in grades 11–12 were more likely to discuss sexual and reproductive health (SRH) issues with their parents or caregivers compared to the students in grades 9–10. This finding aligns with the results of a similar study conducted in Woreta, Ethiopia (30). This might be due to the fact that older students are generally more mature and understand the complexities of SRH issues better, which enables them to approach these topics with greater confidence. By grades 11 and 12, they typically receive a more comprehensive education on SRH, which equips them to ask informed questions and seek guidance. Furthermore, as they age, they become more open and less embarrassed about discussing personal matters, making it easier to talk about SRH (25, 42, 47, 56).

The students who lived with their parents or caregivers were more likely to engage in parent–adolescent communication compared to those who lived alone. This finding aligns with the findings from studies conducted in Debre Markos (42, 57). Living with parents or caregivers provides immediate access to familial support and guidance, which fosters regular interactions and conversations about various topics, including sexual and reproductive health (SRH). This closeness builds stronger bonds and encourages open communication, making adolescents feel more comfortable while discussing sensitive topics such as SRH. Parents or caregivers, feeling a greater sense of responsibility for their children's wellbeing, actively engage in SRH discussions to provide accurate information and guidance. Furthermore, living together allows for greater supervision and monitoring of adolescents' activities, which leads to spontaneous conversations about SRH as parents become aware of their children's concerns. Overall, the presence of parents or caregivers creates a supportive environment conducive to effective parent–adolescent communication about SRH, which promotes better-informed decision-making and healthier behaviors among adolescents (42, 57–59).

The students who had a poor understanding of sexual and reproductive health (SRH) topics were less likely to have parent–adolescent communication on these issues compared to the students with good knowledge. This finding is consistent with the findings of research conducted in Myanmar (60) and Eastern Ethiopia (34). This is because students with good knowledge about SRH topics recognize the importance of seeking guidance on potential risks and consequences associated with sexual behaviors. Their confidence in initiating conversations, coupled with parents' receptiveness to their inquiries, fosters productive dialogue within the family. Furthermore, adolescents with good SRH knowledge actively seek opportunities to discuss these topics with their parents, highlighting the value of parental guidance in making informed decisions about their sexual health. Overall, this emphasizes the critical role of education and open communication in promoting healthy behaviors among young people (43, 61, 62).

The students who had positive attitudes toward sexual reproductive health issues were significantly more likely to have good parent–adolescent communication compared to those with negative attitudes. This finding is similar to the finding of a study conducted in Asella, Ethiopia (44). This could be attributed to the willingness of adolescents to engage in open and honest discussions about sensitive topics related to sexual health. Positive attitudes may indicate a level of comfort and acceptance regarding discussing these issues, which, in turn, fosters effective communication between parents and adolescents. Furthermore, adolescents with positive attitudes may actively seek out information and support from their parents, leading to more frequent and meaningful conversations about sexual reproductive health (44, 63, 64).

### Limitations of the study

The limitations of this study include recall bias and the inability to establish cause-and-effect relationships due to its cross-sectional design. Self-reporting may have been influenced by social desirability bias, especially given the sensitivity of the topic. It is also important to note that not all discussions assumed positive outcomes, as negative and harmful conversations could occur. Furthermore, the study primarily interviewed adults, overlooking valuable perspectives of adolescents that could align or diverge with parental views.

## Conclusion and recommendations

This study revealed a low level of communication between parents and adolescents regarding sexual and reproductive health (SRH). Evidence-based education focusing on SRH topics, such as consent, healthy relationships, communication skills, STDs, contraception, and interpersonal dynamics, is crucial for addressing the low level of communication between parents and adolescents. Implementing peer education among senior students and training teachers in effective communication techniques can enhance parent–adolescent dialogue on SRH. Qualitative research on SRH topics and communication barriers can provide valuable insights for developing interventions.

## Data Availability

The raw data supporting the conclusions of this article will be made available by the authors, without undue reservation.
